# Purinergic Profiling of Regulatory T-cells in Patients With Episodic Migraine

**DOI:** 10.3389/fncel.2018.00326

**Published:** 2018-09-25

**Authors:** Dilyara Nurkhametova, Igor Kudryavtsev, Olga Khayrutdinova, Maria Serebryakova, Rashid Altunbaev, Tarja Malm, Rashid Giniatullin

**Affiliations:** ^1^A.I. Virtanen Institute for Molecular Sciences, University of Eastern Finland, Kuopio, Finland; ^2^Laboratory of Neurobiology, Kazan Federal University, Kazan, Russia; ^3^Department of Immunology, Institute of Experimental Medicine, St. Petersburg, Russia; ^4^Department of Fundamental Medicine, Far Eastern Federal University, Vladivostok, Russia; ^5^Department of Neurology and Rehabilitation, Kazan State Medical University, Kazan, Russia

**Keywords:** purinergic signaling, ATP, adenosine, regulatory T cells, migraine

## Abstract

**Objectives:** Immune responses in migraine are poorly characterized, yet implicated in the disease pathogenesis. This study was carried out to characterize purinergic profiles of T-cells in patients with episodic migraine without aura (MWoA) to provide mechanistic evidence for ATP and adenosine involvement in modulation of immune regulation in migraine.

**Methods:** Peripheral blood samples were obtained from patients with migraine (*n* = 16) and age-matched control subjects (*n* = 21). Subsets of T-cells were identified by flow cytometry based on specific membrane markers.

**Results:** Migraine patients showed reduced total T-cell counts in the peripheral blood. Whereas the total number of CD3+CD4+, CD3+CD8+, or regulatory T lymphocytes (Treg) was not changed, the proportion of Treg CD45R0+CD62L– and CD45R0–CD62L– cells was increased. Interestingly, in migraine, less Treg cells expressed CD39 and CD73 suggesting disrupted ATP breakdown to adenosine. The negative correlations were observed between the duration of migraine and the relative number of CD73+CD39– Tregs and total number of CD73-positive CD45R0+CD62L+ Tregs.

**Conclusion:** Obtained data indicate that T-cell populations are altered in episodic migraine and suggest the involvement of Tregs in the pathophysiology of this disorder. Reduced expression of CD39 and CD73 suggests promotion of ATP-dependent pro-inflammatory and reduction of adenosine-mediated anti-inflammatory mechanisms in migraine.

## Introduction

Migraine is a very common neurological disorder in the modern population. There are two main types of migraine: migraine with aura (MWA) and migraine without aura (MWoA). Migraine aura is presented with ‘recurrent attacks, lasting minutes, of unilateral fully reversible visual, sensory, or other central nervous system symptoms that usually develop gradually and are usually followed by headache and associated migraine symptoms’ (Headache Classification Committee of the International Headache Society (IHS), 2018) ICHD-3. Currently, it is generally accepted that migraine aura is associated with the phenomenon called cortical spreading depression which is a slowly propagating wave of depolarization of brain cells (Somjen, 2001).

Recent evidence suggests the involvement of immune system in the pathogenesis of migraine. Nociceptive trigeminal nerves in dura mater, a likely source of migraine pain (Moskowitz and Cutrer, 1993; Bolay et al., 2002; Olesen et al., 2009; Zakharov et al., 2015), are surrounded by mast cells (Levy, 2009; Kilinc et al., 2017) and T-cells, apparently associated with recently discovered meningeal lymphatic vessels (Aspelund et al., 2015; Louveau et al., 2015). These findings are consistent with local inflammatory reactions leading to neuroinflammation in meninges. The idea of local neurogenic inflammation (sterile inflammation) due to release of neuropeptides from meningeal nerves in migraine was first suggested by M. Moskowitz with colleagues (Moskowitz et al., 1979; Moskowitz and Cutrer, 1993). This view was further developed by many other researchers suggesting involvement of various immune cells and pro-inflammatory substances in development of local meningeal inflammation (Strassman et al., 1996; Reuter et al., 2001, Peroutka, 2005; Levy, 2009; Ramachandran, 2018). In addition, several recent studies have revealed signs of systemic inflammation in migraine. Specifically, the levels of C-reactive protein (CRP) are elevated in serum of migraine patients (Welch et al., 2006; Vanmolkot and de Hoon, 2007). During migraine attacks procalcitonin, another marker of inflammation, is also elevated (Turan et al., 2011). Moreover, a soluble urokinase-type plasminogen activator receptor (suPAR) has been suggested as a prognostic marker for migraine, especially during attack periods. The levels of SuPAR have been reported to be elevated during migraine attack compared to healthy individuals and patients with migraine in the interictal period (Yılmaz et al., 2017).

These data are in line with reported increased levels of several pro-inflammatory cytokines, such as interleukin-1β (IL-1β), IL-6, and tumor necrosis factor-alpha (TNFα) in serum of migraineurs (Sarchielli et al., 2006; Wang et al., 2015; Yücel et al., 2016). Importantly, plasma levels of pro-inflammatory IL-1α and TNFα are elevated not only in adults but also in children with migraine (Boćkowski et al., 2009). At the same time the plasma levels of anti-inflammatory cytokine IL-10, produced by T-cells, are increased during attacks in patients with migraine without aura (Munno et al., 2001). These data indicate that the local (in dura mater) as well as systemic (triggered by cytokines with pleiotropic effects) inflammation are implicated in the pathogenesis of migraine.

The systemic inflammatory reactions are largely mediated by T-cells. T-cells are categorized in subpopulations based on their functional properties and these can be identified based on their expression of specific cell surface markers. From the various T-cell populations, such as helper (CD3+CD4+), cytotoxic (CD3+CD8+) and regulatory (CD3+CD4+CD25high) T cells, the regulatory T cells (Tregs) are specialized in immune suppression and have been recognized as key players in regulation of inflammation (Vignali et al., 2008). Recently the anti-inflammatory capacity of Tregs was suggested to be mediated by purinergic mechanisms and their ability to convert ATP to adenosine via the function of cell-surface ecto-nucleoside triphosphate diphosphohydrolase 1, E-NTPDase1 (CD39), and ecto-5′-nucleotidase (CD73) (Grant et al., 2015; de Oliveira Bravo et al., 2016; Rueda et al., 2016). ATP is known as a powerful pro-inflammatory compound and its concentration is dramatically elevated during inflammation and tissue damage (la Sala et al., 2003). ATP is dephosphorylated by CD39 expressed on Tregs to form ADP/AMP, which is further converted to anti-inflammatory adenosine by CD73. This reaction likely underlies the immunosuppressive effects of Tregs (Takenaka et al., 2016).

Albeit for evidence pointing toward the involvement of T-cells in the pathogenesis of migraine, no studies have analyzed the levels of specific T-cell subtypes in migraine. Here we show for the first time that the level of immunosuppressive Tregs are reduced in migraine patients. Furthermore, we demonstrate that the Treg expression of CD39 and CD73 is reduced suggesting promotion of purinergic mechanisms of inflammation. Our study supports the involvement of systemic inflammatory responses in the disease course of migraine.

## Materials and Methods

### General Description of Patients

Total of 38 patients were assessed for correspondence to inclusion criteria. The criteria were age 18 to 55 years and previously diagnosed migraine without aura, as defined in The International Classification of Headache Disorders-3-beta. Exclusion criteria were oncology, pregnancy, breast-feeding, chronic diseases in stage of decompensation, and migraine with aura. Of initial set of patients, 23 fulfilled inclusion criteria. Twelve patients had oncology, pregnancy or breast feeding, three had migraine with aura, seven declined to participate in the study.

16 patients (all females) were included in the final analysis. **Table 1** provides patients’ detailed demographic characteristics.

**Table 1:** Clinical and demographic characteristics of patients with migraine without aura.

Number of patients	Age	Disease duration (years)	Attacks frequency/per month	Max intensity VAS	Attack duration	Number of days after last attack
1	26	11	8	10	∼24 h	4
2	26	2	2	6	∼24 h	35
3	37	10	2	7	∼48 h	17
4	48	20	3	9	∼72 h	10
5	28	3	1	7	∼24 h	7
6	37	10	2	8	∼24 h	4
7	55	20	4	10	∼24 h	2
8	36	15	3	8	∼48 h	6
9	47	15	2	8	∼24 h	6
10	38	6	0.5	10	∼24 h	28
11	21	6	2	6	∼48 h	5
12	53	20	8	9	∼72 h	2
13	40	10	5	8	∼72 h	1
14	41	11	3	7	∼48 h	7
15	45	20	1	8	∼24 h	14
16	35	6	1	7	∼24 h	12

The data were compared to the results obtained from 21 age-matched healthy female volunteers. Current study was approved by the Medical Ethics Committee of Kazan State Medical University (Kazan, Russia) (protocol No 10 from 20.12.2016). All participants provided written informed consent in accordance with the 2013 Declaration of Helsinki.

### Sample Collection

All experiments were performed on the day of blood collection. Peripheral blood samples were collected into vacuum test tubes containing the K3-EDTA anti-coagulant (cat. 2130122, Weihai Hongyu Medical Devices Co., Ltd, China) and then processed to analyze the frequency of T-cell subsets by using flow cytometry.

### Multicolor Flow Cytometry Assay

The subsets of T cells were analyzed using CytoFLEX and Navios flow cytometers (Beckman Coulter, United States). In brief, 100 μl of peripheral blood was stained with the following cocktail of anti-human antibodies: CD39-FITC (clone A1, cat. 328206, BioLegend, Inc., United States), CD25-PE (clone B1.49.9, cat. A07774, Beckman Coulter, United States), CD62L-ECD (clone DREG56, cat. IM2713U, Beckman Coulter, United States), CD45R0-PC5.5 (clone UCHL1, cat. IM2712U, Beckman Coulter, United States), CD4-PC7 (clone SFCI12T4D11 (T4), cat 737660, Beckman Coulter, United States), CD8-APC (clone B9.11, cat. IM2469, Beckman Coulter, United States), CD3-APC-Alexa Fluor 750 (clone UCHT1, cat. A94680, Beckman Coulter, United States), CD73-Pacific Blue (clone AD2, cat. 344012, BioLegend, Inc., United States) and CD45-Krome Orange (clone J33, cat. A96416, Beckman Coulter, United States). Optimal combinations of antibodies directly conjugated with various fluorochromes were used according to that of previously published (Mahnke and Roederer, 2007). Stainings were carried out in dark at room temperature for 15 min after which red blood cells were lysed by adding 975 μl VersaLyse Lysing Solution (cat. A09777, Beckman Coulter, United States) supplied with 25 μl of IOTest 3 Fixative Solution (cat. A07800, Beckman Coulter, United States) in dark at room temperature for 15 min. The samples were washed once with PBS for 7 min at 330g, resuspended in 250 μl of PBS supplied with 2% of neutral formalin (cat. HT5011-1CS, Sigma-Aldrich Co., United States) and subjected to flow cytometry analysis.

### Gating Strategy of Main T-cell Subsets

**FIGURE 1:**
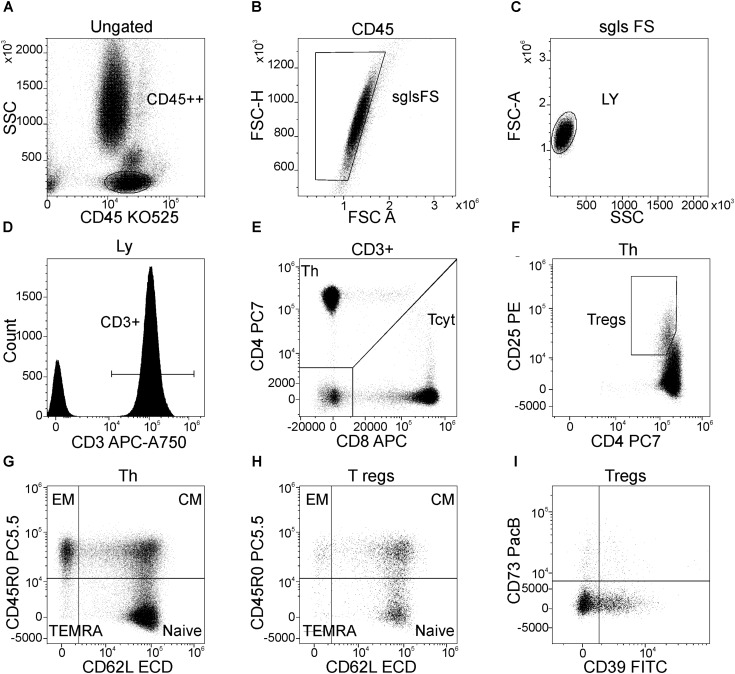
Flow cytometric gating strategy used to identify main peripheral blood T-cell subsets, their differentiation stages, and the expression of CD39 and CD73 by Treg subsets. **(A)** Lymphocytes were selected on the side scatter (SSC) versus CD45 expression plot with a gate “CD45++”; **(B)** Discrimination of lymphocyte doublets, single T-cell gating based on FSC H versus FSC A (the region “sgls FS” is set on single cells); **(C)** All lymphocytes were gated on the side scatter/forward scatter plot with a gate “LY”; **(D)** T-cells were identified from the gate of lymphocytes as “CD3+” within total lymphocyte subset; **(E)** Th (CD3+CD4+) and cytotoxic T-cells (CD3+CD8+) were identified within CD3-positive cells based on CD4 and CD8 expression, respectively; **(F)** Regulatory T-cells (Tregs) were purified as CD4+CD25hi subset within total CD3+CD4+ cells; **(G)** Dot plot representing the expression of CD45R0 and CD62L by Th cells, naïve Th cells are CD45R0–CD62L+, central memory cells are CD45R0+CD62L+, effector memory cells are CD45R0+CD62L– and effector or “terminally-differentiated” Th cells are CD45R0–CD62L– (the same dot plot was used to identify the same differentiation stages of cytotoxic T-cells); **(H)** main differentiation stages of Tregs (the quadrant gate is set at the same position as for total Th cells as well as for cytotoxic T-cells, for that purpose linked quadrant gates were used); **(I)** expression of CD39 and CD73 by total Treg subsets (the same dot plots with the same position of quadrant gates were used when the expression of CD39 and CD73 by naïve, CM, and EM Treg subsets were studied).

Gating scheme used for identification of T cell subsets is shown in **Figure 1**. Helper T (Th) and cytotoxic T cells (Tcyt) were immunophenotyped by using the CD3, CD4, and CD8 antibodies. Th were identified as CD3+CD4+, while Tcyt were CD3+CD8+. Tregs were characterized by the expression of CD3, CD4 and high levels of the IL-2R alpha chain (CD25high). T cell differentiation subsets were defined based on expression pattern of CD45R0 and CD62L as ‘naïve’ (N, CD45R0–CD62L+), central memory (CM, CD45R0+CD62L+), effector memory (EM, CD45R0+CD62L–), and terminally differentiated CD45RA-positive effector T-cells (TEMRA, CD45R0–CD62L–) (Gronert et al., 2015; Ambada et al., 2017). The expression of CD39 and CD73 was evaluated in all above mentioned T-cell subsets. The raw data supporting the conclusions of this manuscript will be made available by the authors, without undue reservation, to any qualified researcher.

### Statistical Analysis

The data were analyzed by using CytExpert Software v.1.1 and Kaluza^TM^ software v.1.5a (Beckman Coulter, United States), Statistica 8 Software (Quest Software Inc., United States) and GraphPad Prism 4 (GraphPad Software, United States). The Shapiro-Wilk’s W test was used to test data for normality. Mann-Whitney U test was employed for comparisons between two different groups of samples. Correlations were evaluated by the Spearman rank correlation test. All data were expressed as mean ± SEM. The level of significance was set as *p* < 0.05.

## Results

### Clinical Description of Patients

The clinical characteristics of patients are shown in **Table 1**. The age of the patients ranged from 21 to 55 years (40.3 ± 2.6). The intensity of pain in most cases was at the level 7–10 (8.0 ± 0.3, visual analog scale (VAS)) with frequency of attacks varying from 0.5 up to 8 per month (2.7 ± 0.6) and disease duration from 2 to 20 years (years from diagnosis made). The patients did not take migraine medications at least two days prior the analysis, except for one patient who used migraine medication one day prior the blood sampling (this patient was excluded from the final analysis).

### Migraine Patients Show Increased Levels of EM and TEMRA Treg Cells

**FIGURE 2:**
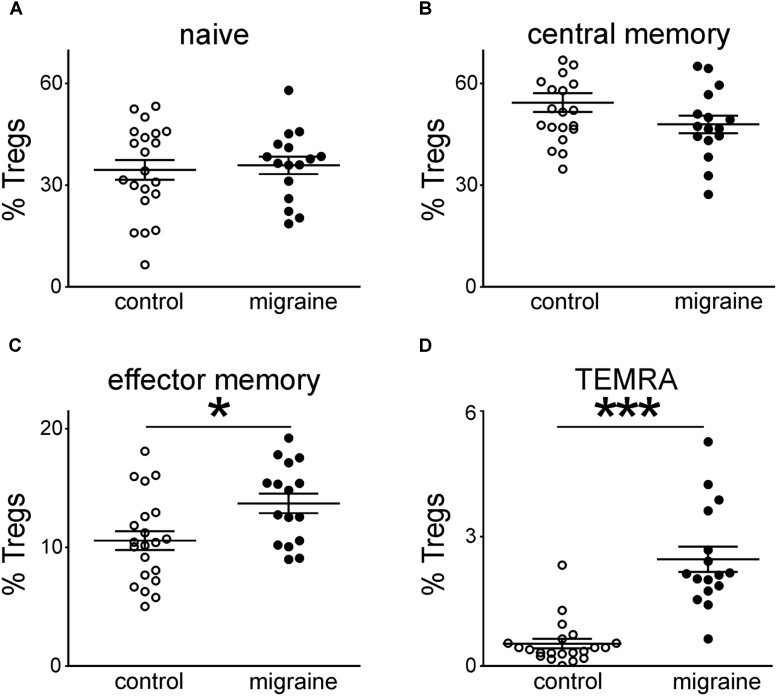
Peripheral blood regulatory T-cells (CD3+CD4+CD25hi) subsets gated on the base of CD45R0 and CD62L expression in healthy controls (white, *n* = 21) and patients with migraine (black, *n* = 16). Percentages of “naïve” (CD45R0-CD62L+) **(A)**, central memory (CD45R0+CD62L+) **(B)**, effector memory (CD45R0+CD62L-) **(C)**, and TEMRA (CD45R0-CD62L-) **(D)** in Tregs cells from peripheral blood. Horizontal lines indicate mean ± SEM. The differences between the groups are shown according to non-parametric Mann-Whitney test (^∗^*p* < 0.05, ^∗∗∗^*p* < 0.001).

**Figure 1** demonstrates the gating strategy for our flow cytometric analysis. Our data revealed that the total CD3+ T cell population was slightly decreased in migraine patients compared to healthy controls (*p* = 0.021, **Table 2**). We further evaluated the percentages of various subsets of T cells that express different patterns of CD45R0 and CD62L (**Figure 2**). Quantitative analysis revealed that the total proportions of Tcyt, Th, and Tregs were not significantly altered in patients with migraine (**Table 2**). However, the percentage of effector memory (EM) and terminally differentiated CD45RA-positive effector (TEMRA) Treg subsets were significantly increased in the peripheral blood from migraine patients (**Table 2** and **Figure 2**).

**Table 2:** The relative number of main T-cell subsets in peripheral blood from healthy volunteers and patients with migraine (percentage of T-cell subsets in total lymphocyte subset, data are mean ± SEM.

Main subsets	Control, %	Migraine, %	p
		
	Mean ± SEM	Mean ± SEM	
**Total T-cells**	77.04 ± 0.98	72.07 ± 1.66	0.021
**Total Tcyt (CD3+CD8+)**	26.1 ± 1.44	23.6 ± 1.43	0.198
‘naïve’ Tcyt (CD45R0–CD62L+)	9.37 ± 1.02	8.75 ± 0.98	0.806
CM Tcyt (CD45R0+CD62L+)	1.46 ± 0.19	1.25 ± 0.18	0.520
EM Tcyt (CD45R0+CD62L–)	6.10 ± 0.69	4.72 ± 0.96	0.061
TEMRA Tcyt (CD45R0–CD62L–)	9.19 ± 0.92	8.92 ± 0.83	0.736
**Total Th (CD3+CD4+)**	47.56 ± 1.25	44.95 ± 1.18	0.177
‘naive’ Th (CD45R0–CD62L+)	22.8 ± 1.65	20.67 ± 1.16	0.481
CM Th (CD45R0+CD62L+)	13.7 ± 0.59	12.85 ± 0.74	0.244
EM Th (CD45R0+CD62L–)	9.61 ± 0.65	9.77 ± 0.41	0.939
TEMRA Th (CD45R0–CD62L–)	1.47 ± 0.33	1.65 ± 0.31	0.141
**Total Treg (CD3+CD4+CD25high)**	2.98 ± 0.17	3.54 ± 0.24	0.177
‘naive’ Treg (CD45R0–CD62L+)	1.09 ± 0.12	1.27 ± 0.11	0.297
CM Treg (CD45R0+CD62L+)	1.56 ± 0.08	1.69 ± 0.16	0.902
EM Treg (CD45R0+CD62L–)	0.32 ± 0.03	0.49 ± 0.05	0.004
TEMRA Treg (CD45R0–CD62L–)	0.019 ± 0.004	0.086 ± 0.011	< 0.001

### Changed Expression of CD73 and CD39 by Treg Subsets in Patients With Migraine

**FIGURE 3:**
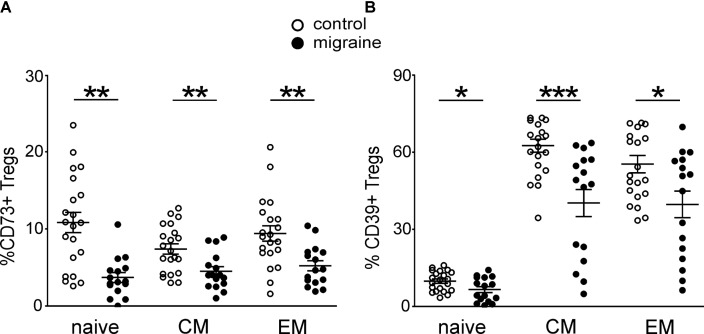
CD73 and CD 39 expression by Treg subsets gated based on CD45R0 and CD62L expression in healthy controls (white, *n* = 21) and migraine patients (black, *n* = 16). **(A)** Percentages of CD73-positive cells within “naïve” (CD45R0-CD62L+), central memory (CD45R0+CD62L+), and effector memory (CD45R0+CD62L-) Tregs. **(B)** Percentages of CD39-positive cells within “naïve” (CD45R0-CD62L+), central memory (CD45R0+CD62L+), effector memory (CD45R0+CD62L-) Tregs. Horizontal lines indicate mean ± SEM. The differences between the groups are shown according to non-parametric Mann-Whitney test (^∗^*p* < 0.05, ^∗∗^*p* < 0.01, ^∗∗∗^*p* < 0.001).

We next characterized the expression of CD73 and CD39 on the peripheral blood Treg subsets at different stages of Treg maturation in migraine patients (**Figure 3**). From the total Treg subset 8.96 ± 0.83% and 42.4 ± 3.13% of the cells were positive for CD73+ and CD39+, respectively, in the control group whereas in patients with migraine the proportion of these cell was significantly lower. Thus, only 4.53 ± 0.56% and 27.6 ± 3.63% of Tregs were CD73 or CD39-positive (*p* < 0.001 and *p* = 0.006, respectively). Our data also show that migraine is associated with reduction of CD73 and CD39 expression in all subsets of Tregs. In healthy controls 10.8 ± 1.32% of the ‘naïve’ CD62L+CD45RO- Tregs were CD73+ and 9.8 ± 0.84% were CD39+, while in patients with migraine these values were significantly lower (3.7 ± 0.64% and 6.5 ± 1.17%, respectively) (**Figure 3**). Similar decrease in the expression of CD39 and CD73 was also observed in CM and EM Tregs (**Figure 3**).

We further measured the number of cells, which express either one of the molecules CD39/CD73 or co-express both of them.

**FIGURE 4:**
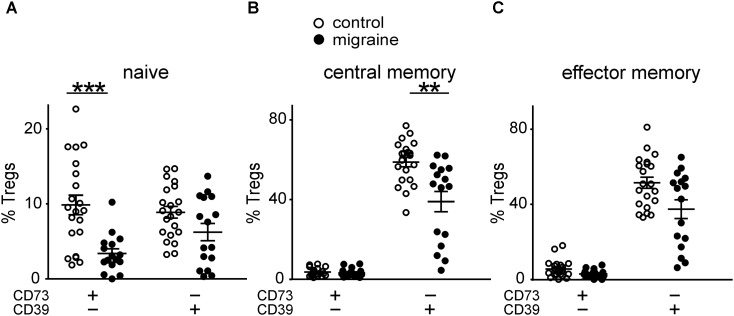
Co-expression of CD39 and CD73 by Treg subsets gated based on the CD45R0 and CD62L expression in healthy controls (white, *n* = 21) and patients with migraine (black, *n* = 16). Percentages of CD73+CD39- and CD73-CD39+ cells within “naïve” (CD45R0-CD62L+) **(A)**, central memory (CD45R0+CD62L+) **(B)**, effector memory (CD45R0+CD62L-) **(C)** Tregs. Horizontal lines indicate mean ± SEM. The differences between the groups are shown according to non-parametric Mann-Whitney test (^∗∗^*p* < 0.01, ^∗∗∗^*p* < 0.001).

Patients with migraine had significantly less CD73+CD39–‘naïve’ Tregs when comparing to control group (3.37 ± 0.64% and 9.86 ± 1.3%, respectively, *p* < 0.001 (**Figure 4A**). The number of CD73–CD39+ cells in ‘naïve’ Tregs tended to be lower in migraine patients (6.23 ± 1.15% versus 8.87 ± 0.78% in control), although this difference did not reach a significance (**Figure 4A**).

No significant differences were found in CD73+CD39– CM Tregs between migraine patients and healthy volunteers (**Figure 4B**). However, the relative number of CD73–CD39+ CM Tregs was significantly higher (*p* = 0.002) in control group (**Figure 4B**).

Co-expression of CD73 and CD39 on effector memory Tregs did not significantly differ between the groups (**Figure 4C**).

**FIGURE 5:**
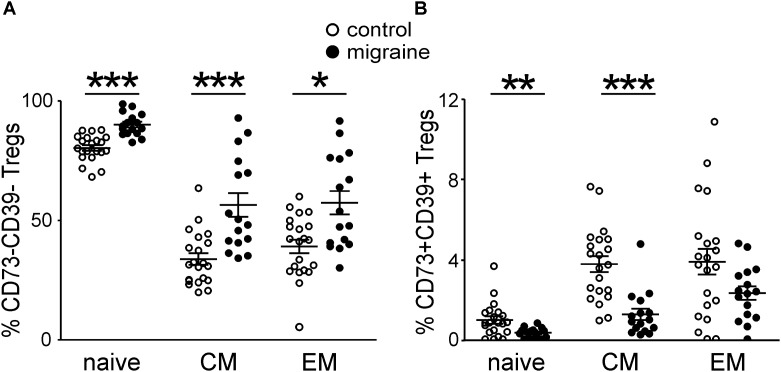
Co-expression of CD39 and CD73 by Treg subsets gated based on the CD45R0 and CD62L expression in healthy controls (white, *n* = 21) and patients with migraine (black, *n* = 16). Percentage of CD73-CD39- **(A)** and CD73+CD39+ **(B)** in “naïve”, central memory, and effector memory Tregs. Horizontal lines indicate mean ± SEM. The differences between the groups are shown according to non-parametric Mann-Whitney test (^∗^*p* < 0.05, ^∗∗^*p* < 0.01, ^∗∗∗^*p* < 0.001).

Patients with migraine had higher levels of Tregs negative for both CD73 and CD39 (**Figure 5A**) and correspondingly lower levels of CD73+CD39+ Tregs (**Figure 5B**).

**FIGURE 6:**
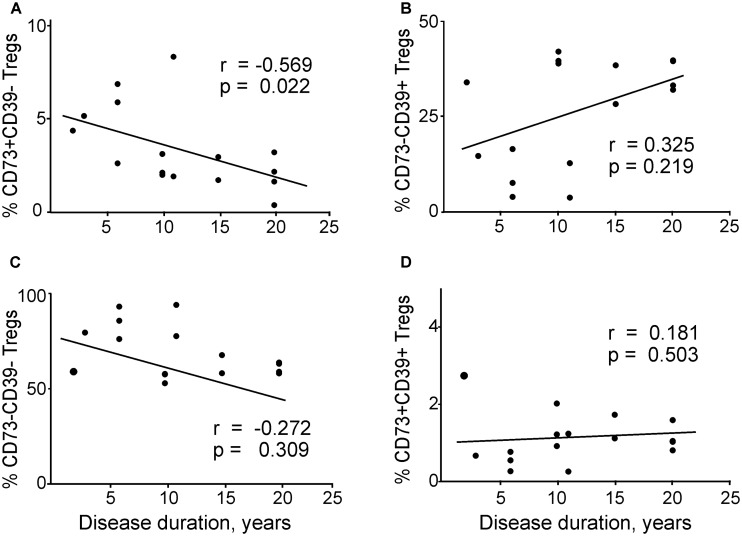
Correlations between disease duration and regulatory T cells expressing different patterns of CD73 and CD39 in patients with migraine according to Spearman rank correlation test: **(A)** CD73+CD39–, **(B)** CD73–CD39+, **(C)** CD73–CD39–, **(D)** CD73+CD39+ in patients with migraine.

Then we analyzed the association between the disease duration and co-expression of CD73 and CD39 in Tregs (**Figure 6**). We found a significant negative correlation between disease duration and CD73+CD39– Tregs (**Figure 6A**). CD73–CD39– subset of general Tregs population also had a tendency to negative correlate with disease duration (**Figure 6C**), however the values did not reach significance. In contrast, CD73–CD39+ and CD73+CD39+ subsets did not correlate with the disease duration (**Figures 6B,D**).

Additionally, duration of migraine negatively correlated with the central memory Tregs subset expressing CD73+, including CD73+CD39- CM Tregs (**Supplementary Figures 1A,B**) but not with expression of CD39+ (**Supplementary Figures 1C,D**). We also explored the correlation between the expression of CD39 and CD73 in Tregs and the number of days after attack (**Supplementary Figure 2**). However, in contrast to the duration of migraine, this analysis failed to reveal significant correlations (*p* > 0.05 for all combinations of CD39 and CD73 expression in Tregs).

## Discussion

Here we describe for the first time changes in Treg subsets and their CD39/73 expression in patients suffering from migraine. The most important finding of our study was that Tregs showed remarkable migraine-associated changes in their expression of CD39 and CD73. These enzymes control the levels of ATP and adenosine - two key purinergic compounds recently emerged as potential modulators of migraine pathology.

Despite accumulating evidence of local and systemic inflammation in migraine, there is only one study of Tregs in this disorder (Arumugam and Parthasarathy, 2016). This study reported that migraine patients, compared to healthy volunteers, exhibited significantly higher percentage of Th cells in their peripheral blood and their Tcyt cell population was significantly reduced (Arumugam and Parthasarathy, 2016). We also observed a trend toward reduced number of Tcyt, however these changes failed to reach statistical significance. Arumugam and Parthasarathy (2016) also suggested the presence of autoimmune response in migraine based on their findings of lower levels of Tregs in patients with migraine. However, purinergic mechanisms including expression of CD39 and CD73 which are important for the function of Tregs (Kobie et al., 2006; Borsellino et al., 2007) were not studied.

Tregs have attracted much attention recently as immune cells capable of controlling excessive inflammation and likely play a crucial role in the maintenance of immune balance (Sakaguchi et al., 2010). There are several mechanisms how Tregs suppress the adaptive immune responses (Vignali et al., 2008). The most well-known is the production of anti-inflammatory cytokines such as IL-10, IL-35 and TGF-β (Grant et al., 2015; Rueda et al., 2016). Tregs can also induce apoptosis of effector T cells through formation of adenosine via CD39/CD73-controlled pathways (Kobie et al., 2006; Borsellino et al., 2007; Grant et al., 2015; Rueda et al., 2016). Notably, the lack of CD39 (Li et al., 2015; Yoshida et al., 2015) or CD73 (Ehrentraut et al., 2013; Wang et al., 2013) may diminish the function of Tregs.

Previous studies showed that calcitonin gene-related peptide (CGRP) – the main mediator of migraine – is involved in the differentiation and polarization of T cells (reviewed by Granstein et al., 2015; Hu et al., 2016). It has been found that CGRP transfected dendritic cells are able to increase the differentiation of CD4+CD25+Foxp3+ Tregs (Matsuda et al., 2012). Whereas this peptide triggers the secretion of pro-inflammatory cytokines from cultured trigeminal glial cells (Thalakoti et al., 2007), in T cells, CGRP promotes expression of the main lineage-specification factor of Tregs, Foxp3 (Rochlitzer et al., 2016; Szklany et al., 2016). Notably, CGRP levels have been reported to be significantly increased in serum of patients with chronic migraine (Ashina et al., 2000; Cernuda-Morollón et al., 2014). In addition, CGRP stimulates the release of ATP in the trigeminovascular system (Yegutkin et al., 2016) and can sensitize ATP receptors in trigeminal neurons (Giniatullin et al., 2008).

Tregs are highly heterogeneous population of peripheral blood lymphocytes and include several subsets. Different classifications of Tregs are used in clinical studies based on their expression of various functional antigens (Sakaguchi et al., 2010; Gronert et al., 2015; Ambada et al., 2017). In our work we identified subsets of Tregs based on their expression of CD45R0 and CD62L, as outlined by previous studies (Sakaguchi et al., 2010; Gronert et al., 2015; Ambada et al., 2017). Out of the four main Treg subsets – ‘naïve’, CM, EM, and TEMRA – we found that the last two were significantly higher in the peripheral blood of migraine patients. Together with CCR7, CD62L is the main antigen responsible for ‘homing’ of T cells to lymphoid tissues. Notably, the latter two Treg subsets lack the expression of CD62L and they have the ability to migrate to peripheral tissues and participate to the suppression of inflammatory reactions. This may be specifically important to migraine-associated neuroinflammation in the meningeal tissues (Pietrobon and Moskowitz, 2013). The changes we observed here in the subsets of Tregs in migraine patients suggest that these reactive cells can contribute to the suppression of migraine-related neuroinflammatory processes in the peripheral tissues.

Another important aspect of this study was the profile of CD39 and CD73, molecules implicated in purinergic signaling. ATP is currently recognized as a key player in triggering neuroinflammatory processes (Burnstock, 2016; Chen et al., 2017) and implicated in migraine pathology (Burnstock, 1981; Magni and Ceruti, 2013; Yegutkin et al., 2016; Zakharov et al., 2016). One key enzyme responsible for the fast deactivation of the pro-nociceptive and pro-inflammatory extracellular ATP is CD39, whereas CD73 is responsible for the transformation of ADP and AMP into anti-nociceptive adenosine (Yegutkin, 2008).

CD39 is expressed in various T cells, including Tregs, and in other effector T helper subsets, such as Th1 and Th17 cells. CD39+ Tregs are more effective than CD39- Tregs in the production of the anti-inflammatory TGF-β in response to activation (Fletcher et al., 2009). Furthermore, CD4+CD25highCD39+ Tregs are effective in suppressing the pro-inflammatory IL-17 implicated in pathological pain (Hung et al., 2017). In contrast, CD4+CD25highCD39- Tregs do not suppress IL-17 secretion (Fletcher et al., 2009). CD39 expression is upregulated in T cells following their infiltration into ATP-rich tissues, such as into tumor microenvironments (Bono et al., 2015). We found that the number of Tregs expressing CD39 was significantly lower in migraine patients, suggesting the accumulation of pro-inflammatory ATP in the extracellular space. This effect was highly significant in the ‘naïve’, CM, and EM Treg subsets. As on potential mechanism for this, we could thus hypothesize that the levels of CD39+CD73+ Tregs were reduced in the systemic circulation because they were recruited to meningeal tissues.

In contrast to high CD39 expression on Tregs, CD73 expression was relatively low. Thus, about 80% in the CD4+CD25high subset were CD39 positive, but only 1–7% of Tregs expressed CD73 on their cell membrane (Mandapathil et al., 2010). Similar to CD39, we found down-regulation in the expression of CD73 in patients’ Tregs. Furthermore, this reduction was present in the ‘naïve’, CM, and EM CD73-positive Tregs. This suggests a reduced level of extracellular adenosine in the peripheral blood of migraine patients and potential ‘disinhibition’ of the inhibitory tone on pain signaling in migraine. The number of Tregs co-expressing both ecto-nucleotidases CD39 and CD73 simultaneously was also significantly lower in patients suffering from migraine. These data indicate that the effective degradation of pro-inflammatory ATP into anti-inflammatory adenosine is disturbed at two sequential stages: ATP → ADP and AMP → adenosine. Impaired effector functions of Tregs in ATP degradation may cause imbalance in the anti- and pro-inflammatory Th subsets, primarily in Th17 cells. Extracellular ATP promotes the synthesis and secretion of pro-inflammatory IL-17, and CD39 on Treg cell surfaces and CD39 ectonucleotidase activity can play a central part in IL-17 down-regulation via ATP removal. For example, during the remission phase of multiple sclerosis, patients could counterbalance Th17 cells that play the main part in neuroinflammation maintenance by triggering an expansion of CD39+ Treg (Peelen et al., 2011). Considering the low expression of CD39 and CD73 in the CD62L-positive subsets of Tregs (‘naïve’ and CM), and the high level of IL-6 in migraine patients (Khaiboullina et al., 2017), it is possible that Th17 cells may mediate Th cell polarization in lymph nodes and mucosa-associated lymphoid tissues. Disrupted ATP homeostasis can also change the functional role of ATP-driven P2X7 receptors implicated in migraine (Gölöncsér and Sperlágh, 2014; Yegutkin et al., 2016) and in triggering the release of the key pro-inflammatory cytokine, IL-1β (Ferrari et al., 2006; Karmakar et al., 2016). The variable CD39/CD73 expression in subsets of Tregs suggests that these cells might differentially participate in different stages of the migraine attack, possibly supporting a pro-inflammatory role of ATP in the initial phase of migraine and later contributing to the termination of the attack via the anti-nociceptive action of adenosine.

In general, our findings show an imbalance in Treg subsets in migraine patients and suggest the inability of Tregs to effectively suppress inflammation in migraine. Further studies should test the contribution of other pro- and anti-inflammatory immune cells (for instance, cytokine-induced killer (CIK) T cells (Horenstein et al., 2018), in migraine pathophysiology, especially in its severe and chronic forms when the inflammatory components are better presented. Several studies have investigated the neuroprotective role of Tregs in various pathological conditions such as stroke, HIV-induced encephalitis and neurodegeneration (Liu et al., 2009; Gong et al., 2011; Li et al., 2013; Huang et al., 2017; Neal et al., 2018). The neuroprotective role is important in the light of recent findings showing that the brains of migrainers may exhibit microinfarctions and microlesions (Colombo et al., 2011; Bashir et al., 2013; Hougaard et al., 2014). Notably, the main migraine mediator CGRP, apart from its’ ability to promote Treg differentiation, is directly neuroprotective in cortical neurons (Abushik et al., 2017) demonstrating a range of previously not appreciated intrinsic defensive mechanisms in migraine.

In summary, we suggest that mobilization of Tregs with functional CD39 and CD73 toward inflammation-affected meninges may be anti-inflammatory and neuroprotective in migraine.

## Author Contributions

DN and IK contributed to data collection, analysis, interpretation, and manuscript writing. OK contributed to patient enrollment, data interpretation, and manuscript writing. MS contributed to data collection and analysis. RA contributed to study design, and patients’ enrollment. TM supervised, wrote, and edited final manuscript. RG contributed to study design and supervision, manuscript writing.

## Conflict of Interest Statement

The authors declare that the research was conducted in the absence of any commercial or financial relationships that could be construed as a potential conflict of interest.
