# Supplementary material for: Purinergic Profiling of Regulatory T-cells in Patients With Episodic Migraine
Source: Front Cell Neurosci. 2018 Sep 25;12:326. doi: 10.3389/fncel.2018.00326 (PMC6167492; doi:10.3389/fncel.2018.00326)
Supplement: Supplementary file 1 [file Data_Sheet_1.PDF]

Supplementary Material

Purinergic Profiling of Regulatory T-cells in Patients with Episodic Migraine

Dilyara Nurkhametova†[1,2], Igor Kudryavtsev†[3,4], Olga Khayrutdinova†[5], Maria Serebryakova[3], Rashid Altunbaev‡[5], Tarja Malm[1], and Rashid Giniatullin*[1,2]

***Correspondence:**
Rashid Giniatullin, MD, PhD
Professor of Cell Biology
A.I. Virtanen Institute University of Eastern Finland
Neulaniementie 2, P.O. Box 1627, 70211 Kuopio, Finland
email: Rashid.Giniatullin@uef.fi
tel: +358403553665
Fax +35817163030

1 Supplementary Data

Duration of migraine negatively correlated with the central memory Tregs subset expressing CD73+, including CD73+CD39- CM Tregs, (Suppl. Figure 1, A and B) but days after last migraine attack does not correlate with expression of different patterns of CD73 and CD39 in Tregs in patients with migraine according to Spearman rank correlation (Suppl. Figure 2).

2 Supplementary Figures and Tables

2.1 Supplementary Figures

Suppl. fig.1

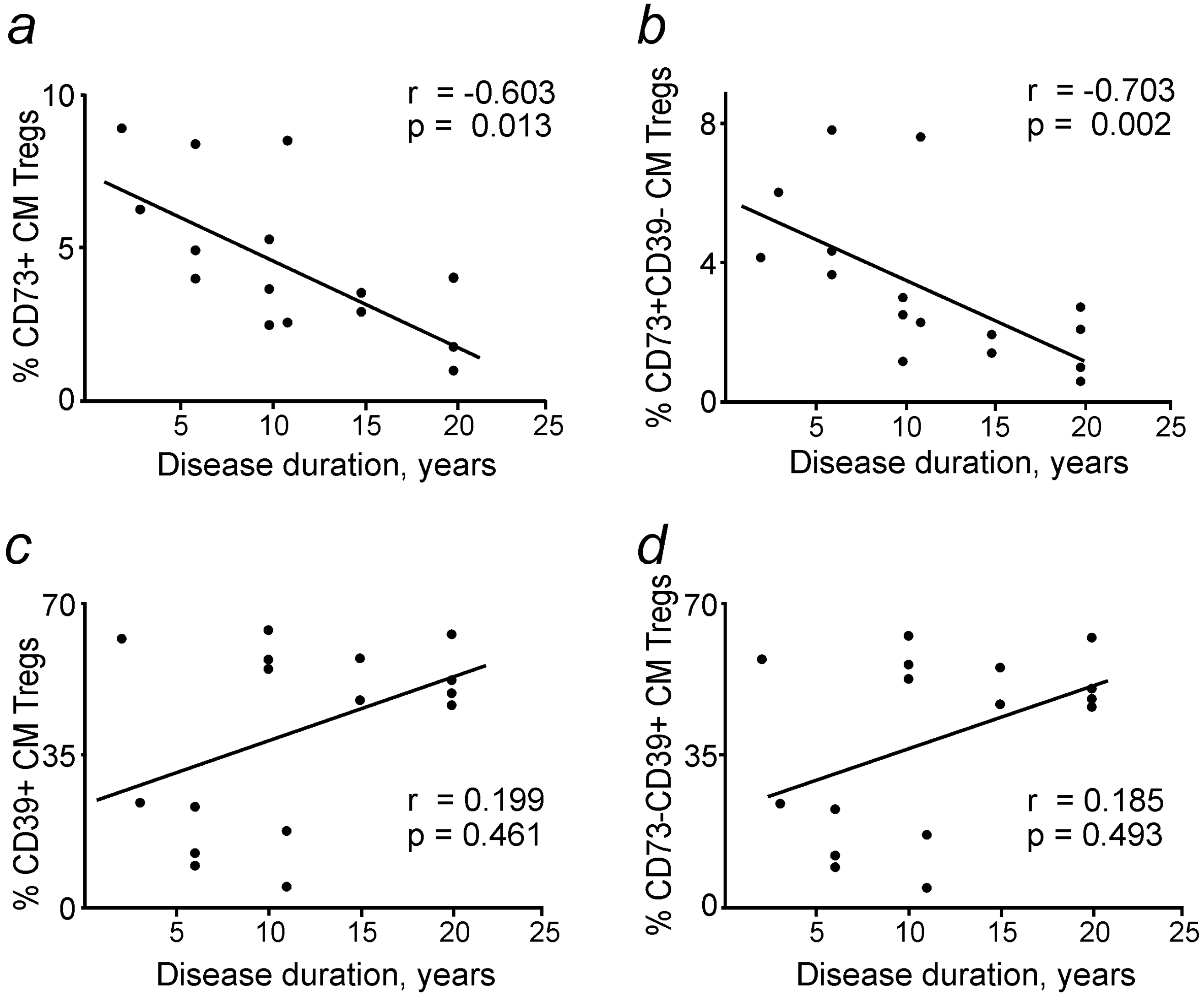

Supplementary figure 1. Correlations between disease duration and central memory Tregs expressing different patterns of CD73 and CD39 in patients with migraine according to Spearman rank correlation test.

Suppl. fig. 2

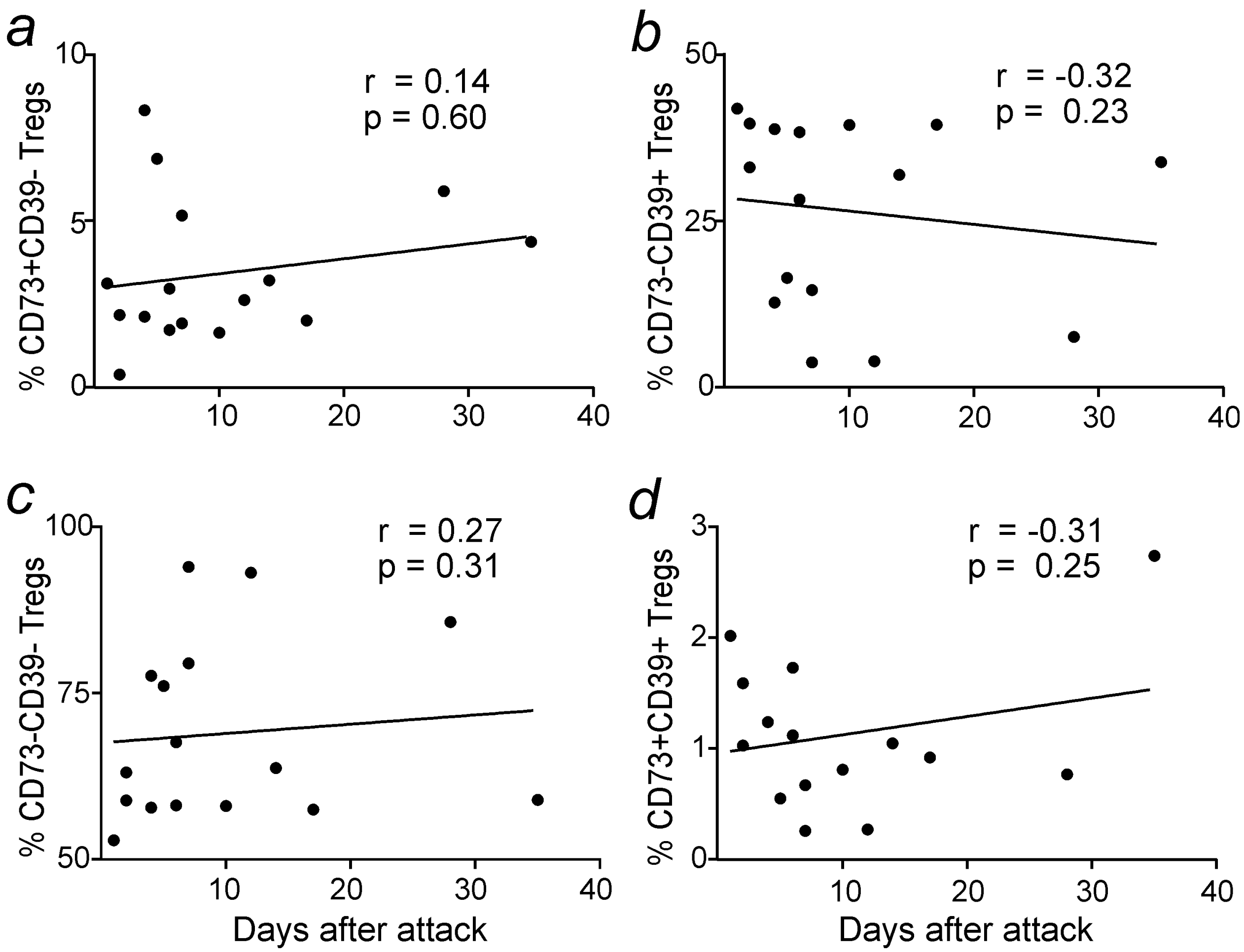

Supplementary figure 2. Correlations between days after last migraine attack and expression of different patterns of CD73 and CD39 in Tregs in patients with migraine according to Spearman rank correlation test.
